# Parallel genetic screens identify nuclear envelope homeostasis as a key determinant of telomere entanglement resolution in fission yeast

**DOI:** 10.1093/g3journal/jkae078

**Published:** 2024-04-25

**Authors:** Rishi Kumar Nageshan, Nevan Krogan, Julia Promisel Cooper

**Affiliations:** Department of Biochemistry and Molecular Genetics, University of Colorado Anschutz Medical Campus, 12801 E. 17th Ave, Aurora, CO 80045, USA; Former address: Telomere Biology Laboratory, Laboratory of Biochemistry and Molecular Biology, NCI, NIH, Bethesda, MD 20892, USA; Department of Cellular and Molecular Pharmacology, University of California, San Francisco, 1700 4th Street, 308D, San Francisco, CA 94158, USA; Quantitative Biosciences Institute (QBI), University of California, San Francisco, CA 94158, USA; J. David Gladstone Institutes, San Francisco, CA 94158, USA; Department of Biochemistry and Molecular Genetics, University of Colorado Anschutz Medical Campus, 12801 E. 17th Ave, Aurora, CO 80045, USA; Former address: Telomere Biology Laboratory, Laboratory of Biochemistry and Molecular Biology, NCI, NIH, Bethesda, MD 20892, USA

**Keywords:** telomere, fission yeast, mitosis, nuclear envelope, chromosome segregation, genome stability

## Abstract

In fission yeast lacking the telomere binding protein, Taz1, replication forks stall at telomeres, triggering deleterious downstream events. Strand invasion from one *taz1Δ* telomeric stalled fork to another on a separate (nonsister) chromosome leads to telomere entanglements, which are resolved in mitosis at 32°C; however, entanglement resolution fails at ≤20°C, leading to cold-specific lethality. Previously, we found that loss of the mitotic function of Rif1, a conserved DNA replication and repair factor, suppresses cold sensitivity by promoting resolution of entanglements without affecting entanglement formation. To understand the underlying pathways of mitotic entanglement resolution, we performed a series of genome-wide synthetic genetic array screens to generate a comprehensive list of genetic interactors of *taz1*Δ and *rif1*Δ. We modified a previously described screening method to ensure that the queried cells were kept in log phase growth. In addition to recapitulating previously identified genetic interactions, we find that loss of genes encoding components of the nuclear pore complex (NPC) promotes telomere disentanglement and suppresses *taz1Δ* cold sensitivity. We attribute this to more rapid anaphase midregion nuclear envelope (NE) breakdown in the absence of these NPC components. Loss of genes involved in lipid metabolism reverses the ability of *rif1*+ deletion to suppress *taz1Δ* cold sensitivity, again pinpointing NE modulation. A *rif1*+ separation-of-function mutant that specifically loses Rif1's mitotic functions yields similar genetic interactions. Genes promoting membrane fluidity were enriched in a parallel *taz1+* synthetic lethal screen at permissive temperature, cementing the idea that the cold specificity of *taz1Δ* lethality stems from altered NE homeostasis.

## Teaser

Pauses in DNA replication can lead to entanglements between telomeres. If these entanglements cannot be resolved at mitosis, genomic instability ensues. Previous studies using the model organism, fission yeast, showed that telomere disentanglement is a particular challenge at moderately low temperatures. Here, a series of genetic screens aimed to identify the regulators of telomere disentanglement. A surprising involvement of factors that control the fluidity of the nuclear membrane in telomere disentanglement emerged.

## Introduction

**Fig. 1.**
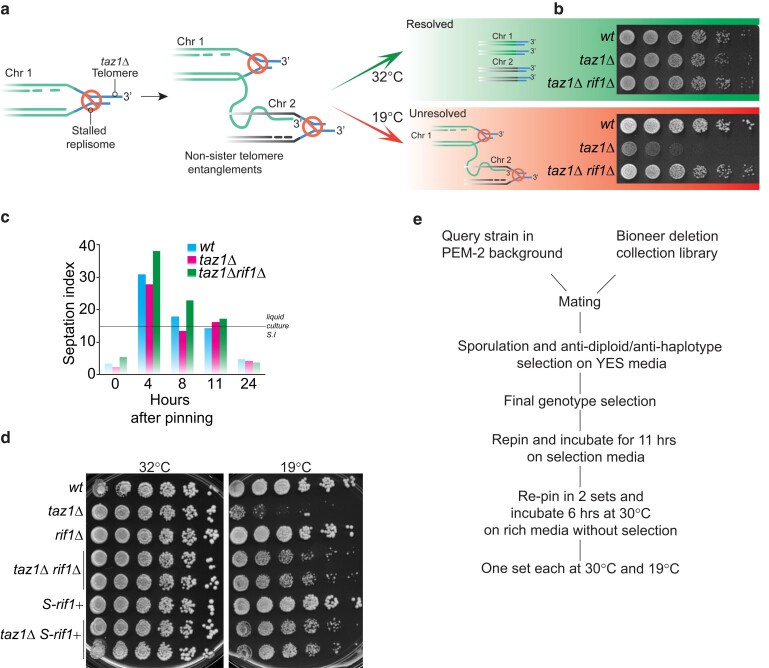
Standardization of protocol followed to identify genes involved in telomere entanglement formation and resolution. a) Representation of telomere entanglement formation in *taz1Δ* cells. The upper and lower arrows show the fates of these entanglements: they are resolved when the cells are grown at 32°C (upper panel, green), but resolution fails when cells are grown at 19°C, causing c/s (lower panel, red). b) Five-fold serial dilution of logarithmically grown cells incubated at 32°C (2 days) or 19°C (7 days) are shown. Rif1 inhibits the resolution of *taz1Δ* telomere entanglements in the cold. c) Standardization of pinning timing to ensure that queried cells are in log phase. *wt* cells from a lawn grown for 16 h were manually pinned onto a fresh YES plate and incubated at 32°C. Septation index was determined by calcofluor staining at the represented time points after pinning. In parallel, a *wt* culture was maintained in log phase (0.5 OD), and the septation index was calculated. The observed septation index (∼15%) is indicated as a dotted line on the graph. d) Five-fold serial dilution of logarithmically grown cells incubated at 32°C (2 days) or 19°C (7 days). All strains were created in the PEM-2 background. e) Summary of the protocol used for the genetic screens. The septation index of a *wt* colony from each plate was manually checked before the plates were moved to their respective temperatures.

Telomeric sequences constrain replisome passage (Miller *et al.* 2006; Bochman *et al.* 2012). In fission yeast, binding of the conserved double-strand telomere binding protein Taz1 promotes replisome passage; an analogous function in promoting telomeric semi-conservative replication is performed by mammalian TRF1, a Taz1 ortholog (Sfeir *et al.* 2009). In the absence of Taz1, stalled telomeric replication forks accumulate and are processed by the RecQ type DNA helicase, Rqh1, rendering them incompetent to resume replication (Rog *et al.* 2009). The resulting irreversibly stalled forks occur at multiple telomeres, resulting in strand invasions between them that form nonsister chromosome entanglements (Fig. 1a; Miller and Cooper 2003; Miller *et al.* 2006; Zaaijer *et al.* 2016). Cycling *taz1*Δ cells maintained at temperatures at or above 25°C successfully resolve these entanglements (Miller and Cooper 2003). However, when grown at ≤20°C (hereafter referred to as “cold”), entanglement resolution fails, causing cold sensitivity (hereafter referred to as “c/s”; Fig. 1b).

To understand the mechanism of entanglement formation, we previously performed an overexpression screen for suppressors of *taz1*Δ c/s (Rog *et al.* 2009). Overexpression of the SUMO deconjugation enzyme Ulp1 or the G1/S transcription factor Cdc10, which controls Ulp1 expression, suppresses *taz1*Δ c/s. These suppressors pointed us toward the action of sumoylated Rqh1, which processes stalled telomeric replication forks to prevent their restart, instead channeling them into entanglement formation in S-phase (Rog *et al.* 2009). The above-described screen left open the question of what factors control entanglement resolution.

The conserved DNA replication and repair protein Rif1 promotes *taz1*Δ c/s (Fig. 1b), as *rif1Δ* confers strong suppression of *taz1Δ* c/s (Miller and Cooper 2003; Zaaijer *et al.* 2016). The loss of Rif1 does not impact telomeric replisome stalling or the formation of entanglements but rather promotes disentanglement. The development of a *rif1*+ separation-of-function (*S-rif1+*) allele allowed us to delineate Rif1's anaphase function specifically as relevant to the resolution of entanglements and suppression of c/s (Zaaijer *et al.* 2016). To address how Rif1 controls the resolution of entanglements and to compile a full array of genes that determine *taz1Δ* viability, we performed a series of genome-wide synthetic genetic array screens to map genetic interactions with *taz1*+ and *rif1*+. To avoid unrelated issues regarding return from stationary phase to mitotic growth in the absence of Taz1, we developed a protocol to keep the cells in log phase throughout all robotic pinning steps. The resulting hits were sorted to select gene deletions that (1) are synthetic sick/lethal with *taz1Δ* alone at the permissive temperature, (2) suppress *taz1Δ* c/s, and (3) reverse *taz1Δ* c/s suppression by *rif1Δ* or *S-rif1+*. The sets of genes thus obtained will inform, respectively, on (1) processes that restrain entanglement formation and/or recapitulate properties conferred by cold temperature, (2) pathways that impact either formation or resolution of entanglements, and (3) pathways that specifically control *taz1Δ* telomere disentanglement in mitosis. Our screening results reveal not only interacting genes from the DNA replication–repair and chromatin organization ontology groups but also genes associated with the nuclear pore complex (NPC) and lipid homeostasis. Collectively, we provide a comprehensive genetic interaction map of the regulators of telomere entanglement resolution and the final steps of mitosis, in which timely nuclear envelope (NE) remodeling plays a surprisingly crucial role.

## Materials and methods

### Media

Media comprised YES broth (Sunrise #2011-500), YES agar (Sunrise #2012-500), and PMG (Sunrise #2060-500). Media and growth conditions were as previously described (Moreno *et al.* 1991). Strains used are listed in Table 1.

**Table 1.** List of strains used in the current study.

Strain no.	Genotype	Source
108	*h- ade6-M210 his3-D1 leu1-32 ura4-D18*	Lab stock
15404	*h+ taz1::hyg ade6-M216 his3-D1 leu1-32 ura4-D18*	Lab stock
15408	*h- taz1::hyg rif1::nat ade6-M his3-D1 leu1-32 ura4-D18*	Lab stock
15323	*h- leu1-32 ade6-210 ura4 D18 mat1_M_cyhS smt0 rp142::cyhR*	Krogan Lab
15328	*h- rif1:nat s-rif1 leu1-32 ade6-210 ura4 D18 mat1_M_cyhS smt0 rp142::cyhR*	Current study
15324	*h- rif1::nat leu1-32 ade6-210 ura4 D18 mat1_M_cyhS smt0 rp142::cyhR*	Current study
15382	*h- taz1::hyg leu1-32 ade6-210 ura4 D18 mat1_M_cyhS smt0 rp142::cyhR*	Current study
15384	*h- taz1::hyg rif1:nat-s-rif1 leu1-32 ade6-210 ura4 D18 mat1_M_cyhS smt0 rp142::cyhR*	Current study
15386	*h- taz1::hyg rif1::nat leu1-32 ade6-210 ura4 D18 mat1_M_cyhS smt0 rp142::cyhR*	Current study
19955	*h- akr1::kan ade6-M210 his3-D1 leu1-32 ura4-D18*	Current study
20017/20018	*h+ taz1::hyg akr1::kan ade6-M his3-D1 leu1-32 ura4-D18*	Current study

### Strain construction

Gene deletions were generated as described previously and used to create further strains through crossing, sporulation, and selection (Bahler *et al.* 1998).

### Synthetic genetic array screening and analysis

We modified the previously described protocol (Roguev *et al.* 2007, 2018) to generate fission yeast genetic interactions maps. Most of the previous genetic screens were performed on stationary phase cells with an endpoint assay, querying viability or sensitivity to environmental challenges. To avoid enrichment for genes that impact cell fitness in or following stationary phase, cells were subjected to a modified protocol. Logarithmically growing *wt* liquid cultures show ∼15–20% septating cells (septation index). We recreated a similar septation index profile by repeated robotic pinning at time intervals short enough to avoid stationary phase and subjected log phase cells to growth at 19°C (Fig. 1c).

Single or double mutants of *taz1Δ*, *rif1Δ*, and/or *S-rif1+* were generated in the PEM-2 strain background as described previously (Roguev *et al.* 2018). We confirmed that the *taz1Δ* c/s and suppression of c/s by *rif1Δ* are unchanged in the PEM-2 background (Fig. 1d). Our screening protocol is outlined in Fig. 1e. Briefly, the query strains were crossed with the Bioneer *Schizosaccharomyces pombe* gene deletion library. Double or triple mutant haploids derived from *taz1Δ*, *rif1Δ*, *S-rif*+, *taz1Δrif1Δ*, or *taz1ΔS-rif1+* were selected. The double mutants were selected on cycloheximide, hygromycin, and G418 and the triple mutants on cycloheximide, hygromycin, G418, and nourseothricin containing media. These selected clones were plated robotically as described earlier with 2 additional rounds of repetitive stamping and a reduced incubation time (as outlined in Fig. 1e) at 30°C on YES plates. The final stamping was on 2 sets of plates. One set was incubated at 30°C and the other at 19°C; both lacked antibiotic selection to minimize any impact on cell fitness. Note that dilution assays to validate the screening results were performed at 32°C as the majority of our previous results have been documented at this temperature; moreover, as mentioned above, at temperatures ≥25°C, *taz1Δ* cells do not show any growth defects. The screen was performed in technical triplicates.

After 3 days of growth, the plates were imaged and growth (colony size) was measured as described earlier (Roguev *et al.* 2018). Normalized growth (NG) values were derived for each colony by normalizing the colony's size to the median colony size on the plate harboring that colony. The NG value was determined for every genotype at both temperatures. The normalized growth ratio (NGR) for a specific genotype was calculated by dividing the NG value at 19°C by the NG value at 30°C, i.e. NGR = NG (19°C)/NG (30°C). The NG conferred by a given gene deletion at a particular temperature reflects the effects of that gene deletion on the growth of the query mutant, while NGR provides information on any cold-specific function of that gene. Log_2_ NG and NGR were also calculated, and the distribution of log_2_ values was plotted to determine the median and SDs, thus identifying the outliers, i.e. the genes conferring strong genetic interactions. Outliers were assigned by either compiling the top 25th percentile or using the 2 SD method.

**Fig. 2.**
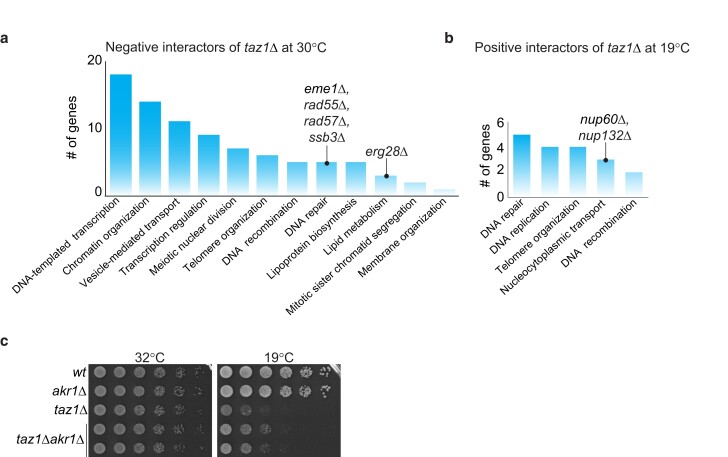
Genes involved in telomere entanglement formation, resolution, and cold sensitivity. a) Graph of number of genes from the respective GO groups that show negative genetic interactions with *taz1*Δ at 30°C. b) Graph of number of genes from the respective GO groups that show positive interactions with *taz1*Δ cells at 19°C. Note: only selected groups are represented here; an unedited GO tag enrichment list is provided in Sheet 3 of Supplementary Tables 2 and 3 for a) and b), respectively. c) Five-fold serial dilution of logarithmically growing cells incubated at 32°C (2 days) or 19°C (7 days).

*rif1Δ* emerged as a *taz1Δ* suppressor in the top 25th percentile. The top 25th percentile of genetic interactors with *rif1Δ*, *S-rif1+*, *taz1Δrif1Δ*, and *taz1ΔS-rif1+* were further analyzed to select hits that restored *rif1+* mitotic function by promoting *taz1Δ* c/s without affecting the respective single mutants. Gene ontology (GO) enrichments were determined using www.pombase.org (biological process slim) (The Gene Ontology Consortium 2018; Rutherford et al. 2024) inbuilt GO tags. Supplementary Table 1 provides the unprocessed list of the top 25th percentile genes that show genetic interactions with the specified query strains, their corresponding NGR along with log_2_ NGR values, and their gene information. The log_2_ NG at 30°C was directly used as the measure of interaction (as with *taz1Δ* in Fig. 2a). Supplementary Table 2 displays data derived by the 2 SD method along with GO analysis for genes showing negative interaction specifically with *taz1Δ* at 30°C.

## Results and discussion

To investigate the mechanisms that control *taz1Δ* entanglement resolution, we performed a series of parallel screens in which the *S. pombe* gene deletion library was crossed with *taz1Δ*, *rif1Δ*, and *taz1Δrif1Δ* cells to identify those gene deletions that rescue or exacerbate *taz1Δ* c/s or reverse the suppression of c/s conferred by *rif1+* deletion. We also employed the *S-rif1+* allele, which acts as a null specifically for Rif1's mitotic functions, in parallel screens to distinguish those factors that act at S- vs M-phase (Zaaijer *et al.* 2016). Based on our previous work, we expected a number of genetic interactions with *taz1Δ*. For instance, our previous studies showed that *rap1+* deletion confers synthetic sickness with *taz1Δ* at 19°C, while *ulp2Δ* and *rif1Δ* are suppressors of *taz1Δ* c/s; these interactions appeared in our screens (Supplementary Table 1), validating their efficacy (Miller and Cooper 2003).

### Replication fork processing factors are required for *taz1*Δ cell viability

To identify the players that control *taz1*Δ cell viability at permissive temperature, we ascertained specific genetic interactions with *taz1*Δ by filtering out any hits that show similar interaction with *rif1*Δ (Supplementary Table 2). GO analysis of the filtered hits revealed genes involved in transcription as the top interactors, followed by factors controlling chromatin organization. These factors could change the chromatin landscape of *taz1*Δ telomeres or may act indirectly via controlling expression of other genes (Fig. 2a).

The stalled replication forks at *taz1Δ* telomeres are processed by DNA replication and repair factors such as Rqh1 (Rog *et al.* 2009), which promotes the aberrant processing of these forks to form entanglements. The gene encoding Eme1, a component of an endonuclease complex that promotes homologous crossovers, is required for full *taz1*Δ cell viability, as are the DNA replication–repair factors Rad55, Rad57, and the Replication Protein complex A subunit Ssb3. It will be important to dissect whether these factors act at the level of entanglement generation, mitotic entanglement resolution, or suppression of nonhomologous end-joining in G2 cells (Ferreira and Cooper 2001, 2004).

### Loss of NPC components suppresses *taz1*Δ c/s

Several of the strongest suppressors of *taz1*Δ c/s encode components of NPCs (Fig. 2b; Supplementary Table 3). We have validated the positive genetic interactions with *nup60*Δ and *nup132*Δ and performed an extensive functional analysis (Nageshan *et al.* 2024). This analysis pinpointed the functions of Nup60 and Nup132 in a local NE breakdown event as crucial to the resolution of *taz1Δ* telomeric entanglements. Briefly, fission yeast undergoes NE breakdown in a temporally and spatially restricted manner, at the anaphase midregion (Dey *et al.* 2020; Exposito-Serrano *et al.* 2020), across which any persisting chromosome entanglements are stretched. The exposure of the spindle to the cytoplasm afforded by this NE breakdown is essential for spindle disassembly and successful nuclear division. The anaphase midregion NE harbors NPCs that are sequentially dismantled, leading to the local NE breakdown. Loss of NPC components hastens this process, ensuring a fragile midregion (Nageshan *et al.* 2024). Along with our analysis of key hits in the parallel screen for reversal of suppression by *rif1Δ* (see below), our data show that advancing NE breakdown promotes telomere entanglement resolution.

### Lipid membrane homeostasis regulates entanglement resolution

Genes that control lipid biosynthesis and membrane homeostasis (Fig. 2a) emerged as prominent genetic interactors with *taz1Δ*. In mammalian cells, cholesterol controls membrane fluidity, with higher levels of cholesterol generally conferring greater fluidity; ergosterol carries out these functions in fungal membranes. The gene encoding Erg28, which is involved in ergosterol biosynthesis, is required for *taz1*Δ cell survival even at permissive temperature (30°C; Fig. 2a; Supplementary Table 2). Budding yeast cells lacking Erg28 have been shown to harbor a 3-fold reduction in ergosterol along with the accumulation of 3-keto sterols and carboxylic acid sterols and, therefore, display reduced membrane fluidity (Gachotte *et al.* 2001; Mo and Bard 2005). This genetic interaction suggests that membrane fluidity promotes resolution of telomere entanglements and is consistent with our observation that treatment with membrane-fluidizing agents suppresses *taz1Δ* c/s (Nageshan *et al.* 2024). We hypothesize that enhanced NE rigidity mimics cold temperatures, which increase phospholipid membrane rigidity. In either cold conditions or at 30°C in the absence of Erg28, NE fluidity is reduced, confounding entanglement resolution by inhibiting anaphase midregion NE breakdown.

In contrast to the synthetic sickness between *erg28Δ* and *taz1Δ*, deletion of the gene encoding the Akr1 palmitoyl acyltransferase confers a mild suppression of *taz1Δ* c/s. A recent report showed that Akr1 localizes to the nuclear periphery as well as the cell tips and is required for fusion of 2 NEs during meiosis (Pham *et al.* 2023); in the absence of Akr1, karyogamy fails. We generated a new double mutant by deleting *akr1*+ in the *taz1Δ* background to avoid the need to undergo meiosis and confirmed c/s suppression of *taz1Δ* by *ark1Δ* (Fig. 2c; Supplementary Table 3). Though we do not yet know the relevant palmitoylation targets, it is tempting to speculate that Rif1 might be a target. Previous reports have demonstrated that budding yeast Rif1 is targeted to the inner NE via palmitoylation (Park *et al.* 2011; Fox and Gartenberg 2012; Fontana *et al.* 2019); such targeting facilitates the ability of Rif1 to direct DNA double-strand break repair away from resection-dependent homologous recombination and toward nonhomologous end-joining (Fontana *et al.* 2019), neither of which controls *taz1Δ* telomere entanglements (Miller and Cooper 2003; Rog *et al.* 2009). Hence, while these previous studies focused on Rif1's role in S-phase or interphase, palmitoylation may also play a role in Rif1's mitotic localization and actions.

### Loss of genes involved in lipid metabolism restores Rif1's anaphase function in regulating telomere entanglement resolution

**Fig. 3.**
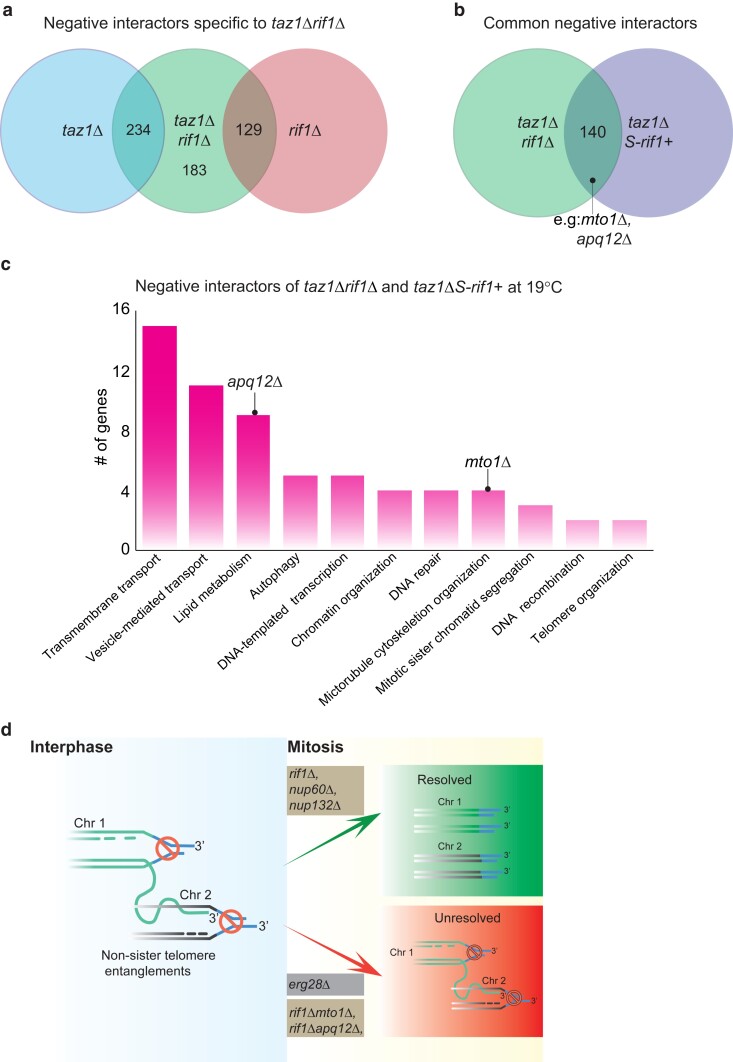
Genes that revert the ability of *rif1*Δ to suppress *taz1Δ* anaphase phenotype. a) Venn diagram of the genes that show negative interactions with *taz1*Δ*rif1*Δ, specifically in the top 25th percentile with respect to the strength of the reversion. b) Venn diagram of interactors common to *taz1Δrif1Δ* and *taz1ΔS-rif1*+. The common genes specifically restore the anaphase Rif1 function that is relevant to *taz1Δ* telomere entanglement resolution. c) Graph of number of genes from selected GO groups that show negative interactions with *taz1*Δ*rif1*Δ and *taz1*ΔS-*rif1*+ cells at 19°C. An unedited summary of the GO term analysis is provided in Sheet 3 of Supplementary Table 4. d) Representation of telomere entanglement as in Fig. 1a with the addition of genetic interactions observed herein. Gene deletions that enable resolution are above the green arrow, while those that hinder resolution are below the red arrow. Genotypes that are highlighted in beige represent interactions at 19°C, and those highlighted in gray represent interactions at 32°C.

To delineate the molecular mechanisms of Rif1's anaphase function, we screened for gene deletions that specifically revert the suppression of *taz1Δ* c/s by *rif1*Δ (Fig. 3a and b). We also carried out the analogous screen for reversion of *taz1Δ* c/s in a *S-rif1*+ background in which Rif1's S-phase functions are retained while its mitotic function is lost (Zaaijer *et al.* 2016). GO analysis of the gene deletions that reverted both *rif1*Δ and *S-rif1+* phenotypes show lipid metabolism as one of the most prominent ontology groups (Fig. 3c; Supplementary Table 4). Among these, *apq12+* loss reverted Rif1's mitotic function, conferring c/s to *taz1Δrif1Δ* cells without affecting the c/s of single *taz1Δ* mutants. Budding yeast Apq12 is a NE and endoplasmic reticulum protein whose loss leads to defects in NPC assembly stemming from deregulation of NE lipid homeostasis (Scarcelli *et al.* 2007; Schneiter and Cole 2010). The defects in NPC assembly in *apq12Δ* cells are averted by treatment with low concentrations of the membrane-fluidizing agent benzyl alcohol (BA). In fission yeast, *apq12*Δ cells have likewise been shown to suffer defects in NE homeostasis (Tamm *et al.* 2011). Altered NE dynamics in the absence of Apq12 would be compatible with a model in which more rapid NPC disassembly, afforded by Rif1 loss, is counteracted by severe levels of NE rigidity in *apq12Δ* cells at 19°C, delaying anaphase midregion NE breakdown and reverting *taz1Δ* viability.

Our analyses also pinpointed a gene not previously implicated in NE homeostasis, *mto1+*, as a key player in the dynamics of anaphase midregion NE breakdown. Mto1 emerged as a gene product whose loss strongly reverts the suppression of *taz1Δ* c/s by *rif1+* deletion (Fig. 3c; Supplementary Table 4). We confirmed and analyzed this genetic interaction (Nageshan *et al.* 2024). Mto1 has been known as a cytoplasmic microtubule-organizing protein whose loss leads to the disappearance of several classes of cytoplasmic microtubules, generating high concentrations of free tubulin and, in turn, spindle persistence (within the nucleus; Sawin *et al.* 2004). However, we found that *mto1Δ* cells also suffer delayed anaphase midregion NE breakdown, possibly stemming from the loss of cytoplasmic MTs that physically prod the anaphase midregion NE and stimulate its disassembly (Nageshan *et al.* 2024). Our analysis included separation of Mto1's spindle persistence and NE breakdown-promoting functions and demonstrated that Mto1 promotes entanglement resolution via advancement of anaphase midregion NE breakdown.

### Vesicle-mediated transport process regulates telomere disentanglement

Gene deletions that have been classified to function in vesicle-mediated transport show negative genetic interactions with *taz1Δ* at 30°C (Fig. 2a) and with *taz1Δrif1Δ* at 19°C (Fig. 3c). Genes (*vsp26+*, *sft1*+, and *tlg2*+) encoding proteins that are involved in transport of vesicles between the ER and Golgi apparatus, along with a gene (*dot2*+) encoding an ESCRTII complex subunit, were required for *taz1Δ* viability at 30°C. Similarly, loss of *sft2+*, *psg1+*, or SPAC9ES.04 reversed suppression of *taz1Δ* c/s by *rif1Δ*. A recent report presents a comprehensive physical interaction screen between fission yeast NPCs and the inner NE proteins Ima1 and Lem2, using the yeast 2-hybrid approach (Varberg *et al.* 2020). This study found that many of the proteins encoded by the abovementioned genes interact with inner NE proteins; for instance, Ima1 interacts with Sft1, Sft2, Psg1, and SPAC9ES.04p. Additionally, Cut11, a component of both the NPC and the spindle pole body, shows interactions with this set of gene products. These interactions highlight a functional interplay between NE dynamics, genes classified as involved in vesicle transport, and *taz1Δ* telomere disentanglement. These observations herald an exploration of how chromosome entanglements in the anaphase midregion communicate with the NE breakdown machinery and how membrane sorting/repair proteins participate in this process.

Collectively, the synthetic genetic array screens hold a wealth of information, prominently suggesting unforeseen links between DNA processing events and regulators of NE fluidity and NPC disassembly (Fig. 3d). The synthetic sickness between *erg28Δ* and *taz1Δ* suggests that membrane rigidity is a key factor in mediating the cold specificity of *taz1Δ* lethality. Additional genes whose functions have been implicated in NE fluidity or NPC dismantlement-induced anaphase NE breakdown play crucial roles in *taz1Δ* telomere entanglement resolution. Moreover, in-depth analyses of genes classified in GO groups seemingly unrelated to NE breakdown may uncover roles in this process, as has been the case for Mto1 (Nageshan *et al.* 2024). A comprehensive analysis of the DNA repair/replication/recombination genes identified will also be crucial for dissecting the mechanisms that make NE breakdown, and therefore cytoplasmic exposure of telomere entanglements, so critical for their resolution.

## Data Availability

All the relevant data referred to in this article are available in the supplemental material: https://doi.org/10.25387/g3.25536280. Strains used in the current study are available upon request.
